# Impairment of rigidity sensing caused by mutant *TP53* gain of function in osteosarcoma

**DOI:** 10.1038/s41413-023-00265-w

**Published:** 2023-05-29

**Authors:** Ming Luo, Mingyang Huang, Ningning Yang, Yufan Zhu, Peng Huang, Zhujun Xu, Wengang Wang, Lin Cai

**Affiliations:** 1 Department of Orthopedics, Zhongnan Hospital of Wuhan University, Wuhan, 430071 China; 2 Department of Orthopedics, The First Affiliated Hospital of Zhengzhou University, Zhengzhou, 450052 China; 3 Department of Emergency, The First Affiliated Hospital of Zhengzhou University, Zhengzhou, 450052 China

**Keywords:** Bone cancer, Bone cancer

## Abstract

Osteosarcoma (OS) is the most common primary malignant pediatric bone tumor and is characterized by high heterogeneity. Studies have revealed a wide range of phenotypic differences among OS cell lines in terms of their in vivo tumorigenicity and in vitro colony-forming abilities. However, the underlying molecular mechanism of these discrepancies remains unclear. The potential role of mechanotransduction in tumorigenicity is of particular interest. To this end, we tested the tumorigenicity and anoikis resistance of OS cell lines both in vitro and in vivo. We utilized a sphere culture model, a soft agar assay, and soft and rigid hydrogel surface culture models to investigate the function of rigidity sensing in the tumorigenicity of OS cells. Additionally, we quantified the expression of sensor proteins, including four kinases and seven cytoskeletal proteins, in OS cell lines. The upstream core transcription factors of rigidity-sensing proteins were further investigated. We detected anoikis resistance in transformed OS cells. The mechanosensing function of transformed OS cells was also impaired, with general downregulation of rigidity-sensing components. We identified toggling between normal and transformed growth based on the expression pattern of rigidity-sensing proteins in OS cells. We further uncovered a novel *TP53* mutation (R156P) in transformed OS cells, which acquired gain of function to inhibit rigidity sensing, thus sustaining transformed growth. Our findings suggest a fundamental role of rigidity-sensing components in OS tumorigenicity as mechanotransduction elements through which cells can sense their physical microenvironment. In addition, the gain of function of mutant *TP53* appears to serve as an executor for such malignant programs.

## Introduction

Osteosarcoma (OS) is the most common primary malignant bone tumor in children and adolescents, with a 5-year survival rate of less than 20% following the development of metastases.^1,2^ Despite advancements in oncologic diagnostics and therapies, improved clinical outcomes are severely hindered by the intrinsic heterogeneity of OS, which is thought to be linked to its metastatic propensity and chemoresistance.^3^ Previous studies that tested different OS cell lines identified distinct phenotypes in terms of in vivo tumorigenicity and in vitro colony-forming capacity.^4^ The reliability of experimental findings may be affected by the selection of the appropriate cell line for OS-related investigations. However, the mechanism underlying the heterogeneity among OS cell lines is still unclear.

Matrix stiffness is one of the most critical components of mechanotransduction for appropriate development in normal cells. Complex cellular mechanosensing activities are required to generate appropriate growth signals for survival, growth, or death when cells attach to the extracellular matrix (ECM).^5^ The function of rigidity-sensing components is dictated mainly by kinases (e.g., EGFR, HER2, ROR2, and AXL) and cytoskeletal proteins (e.g., MYH9, TPM1, TPM2, TPM3, FLNA, ACTN1, and ACTN4).^6–8^ During normal cell growth, the process of anoikis is activated when cells lose connection to nearby supports.^9^ Transformed growth or anoikis resistance refers to a state in which malignant tumor cells can evade context-dependent conditions and survive.^10^ Earlier studies have suggested that the absence of normal signaling produced by attachment to the ECM could lead to anoikis resistance in human OS cells.^11^ However, it remains unclear whether alterations in rigidity-sensing elements exist in OS cell lines or contribute to tumorigenicity.

As a transcription factor, the wild-type TP53 protein directly binds to a certain DNA sequence and regulates hundreds of genes to control diverse cellular responses that collectively prevent tumorigenesis. The *TP53* gene is mutated in approximately half of all human malignancies, and most mutations result in a single amino acid substitution within the DNA-binding domain.^12,13^ Three cancer-causing attributes of mutant *TP53* have been postulated: the inability to activate target genes for tumor suppression (loss-of-function), the absence of the blocking function of wild-type *TP53* during the early transformation stage (dominant-negative effects), and the presence of oncogenic characteristics to promote cancer cell proliferation and apoptosis escape (gain-of-function, GOF).^13^ The *TP53* mutation frequency in OS patients is currently believed to range from 47 to 90%, much higher than initially determined.^14,15^ Moreover, a meta-analysis including 210 OS patients from eight trials revealed that *TP53* mutations had an unfavorable effect on overall survival.^16^ Loss of the ability to form active rigidity-sensing modules has been observed in many malignancies, including human breast cancer, human fibrosarcoma, and mouse lung carcinoma.^6^ In mantle cell lymphoma, downmodulation of genes involved in actin cytoskeleton organization and cell adhesion was associated with the TP53 pathway.^17^ Mutant *TP53* was found to increase the invasion and metastatic activity of human non-small cell lung cancer cells via EGFR/integrin signaling.^18^ The role of mutant *TP53* in different OS cell lines is currently unclear, and its contribution to tumorigenicity through controlling rigidity sensing remains unknown.

We examined tumorigenicity, anoikis resistance, and the mechanosensing function across OS cell lines and detected anoikis resistance in transformed OS cells. Interestingly, the mechanosensing function of transformed OS cells was impaired, with general downregulation of rigidity-sensing proteins. Furthermore, we identified toggling between normal and transformed growth based on the expression pattern of rigidity-sensing components in OS cells. Instead of the mechanosensor protein YAP1, the GOF mutant of *TP53* (R156P) played a critical role in controlling the rigidity-sensing function of malignant OS cells. Our findings provide new insights into the role of mutant *TP53* in inhibiting rigidity sensing and suggest that the loss of mechanosensing is a critical factor in sustaining the tumorigenicity of OS cells.

## Results

### Different anoikis resistance among OS cell lines

**Fig. 1:**
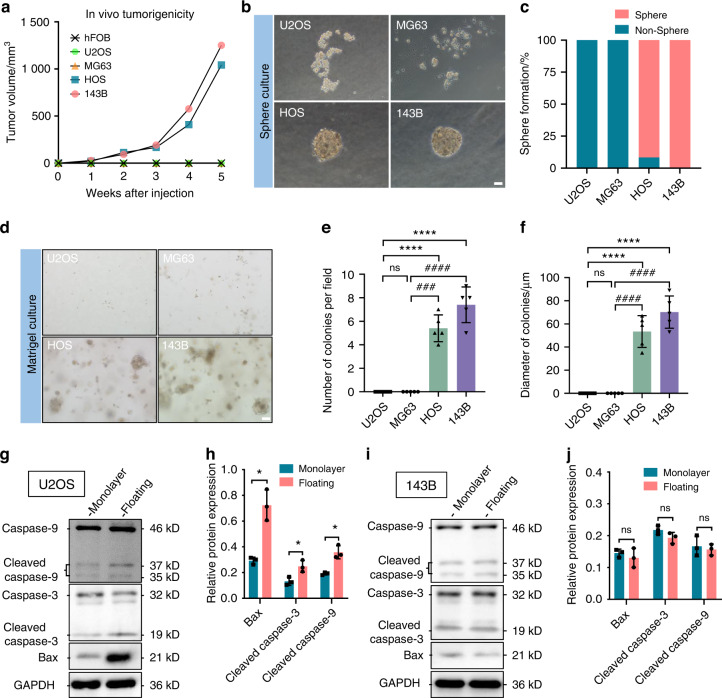
Different levels of resistance to anoikis among osteosarcoma cell lines. **a** The volumes of tumors derived from different osteosarcoma cell lines in a subcutaneous implantation tumor model. *n* = 5 in each group; the data are presented as the means. Sphere cultures of U2OS, MG63, HOS, and 143B cells propagated for 7 days (**b**) and quantification (**c**). *n* = 12 in each group; the data are presented as the means. 3D cultures of U2OS, MG63, HOS, and 143B cells with Matrigel for 2 weeks (**d**) and quantification (**e**, **f**). *n* = 5 in each group; the data are presented as the mean ± s.d. values. Representative Western blots of anoikis-related proteins in U2OS cells in monolayer or floating culture for 3 days (**g**) and quantification (**h**). *n* = 3 in each group; the data are presented as the mean ± s.d. values. Representative Western blots of anoikis-related proteins in 143B cells in monolayer or floating culture for 3 days (**i**) and quantification (**j**). *n* = 3 in each group; the data are presented as the mean ± s.d. values. The graphs show the individual data points derived from three independent measurements and the means. *P < 0.05; ****P < 0.000 1; ^###^*P* < 0.001; ^####^*P* < 0.000 1. Scale bars, 50 μm

We began our analysis with the xenograft model to investigate the tumorigenicity of U2OS, MG63, HOS, and 143B cells. HOS and 143B cells rapidly formed tumors that grew to a volume of 1 000 mm^3^ within 5 weeks, whereas U2OS and MG63 cells did not form tumors (Fig. 1a). We next tested the tumorigenicity of OS cell lines in vitro with a sphere formation assay. HOS and 143B cells formed spheres after suspension culture for 7 days, but no visible spheres were observed in the U2OS and MG63 cell cultures (Fig. 1b, c). We also established a 3D culture model with Matrigel, a mouse tumor extracellular matrix protein mixture. Consistent with the results of the in vivo tumorigenicity and in vitro sphere formation assays, we found that HOS and 143B cells could form spherical colonies in Matrigel, but U2OS and MG63 cells could not (Fig. 1d–f). Anoikis is caused by the disruption of cell-matrix interactions in normal cells, and floating culture is a classical method to induce anoikis that has been reported in many previous studies.^19–22^ To explain the discrepancy in tumorigenicity, we quantified the expression levels of anoikis-related proteins in OS cells under attachment loss conditions. Compared to monolayer culture conditions, we observed increased protein levels of BAX, cleaved Caspase-3, and cleaved Caspase-9 in U2OS cells after floating culture for 3 days, whereas no differences in these levels were found in 143B cells (Fig. 1g–j). Thus, tumorigenicity differs markedly among OS cell lines, and anoikis resistance was uncovered in malignant OS cell lines (143B and HOS cells).

### Impaired rigidity sensing function in transformed OS cell lines

**Fig. 2:**
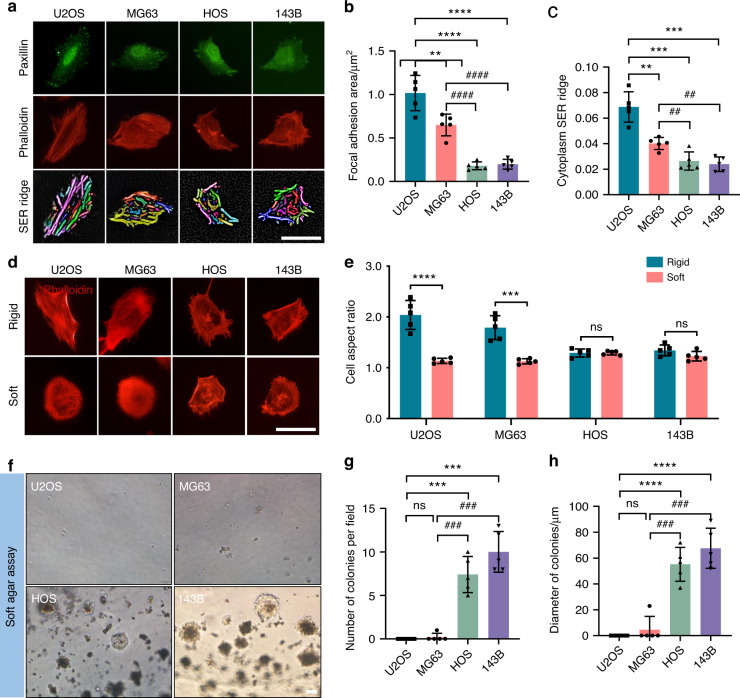
Different rigidity sensing functionality among osteosarcoma cell lines. Staining for paxillin (green) and actin (red) in U2OS, MG63, HOS, and 143B cells cultured on glass surfaces overnight (**a**) and quantification (**b**, **c**). *n* = 5 fields in each group; the data are presented as the mean ± s.d. values. Staining for actin (red) in U2OS, MG63, HOS, and 143B cells cultured on rigid (40 kPa) or soft (4 kPa) hydrogel surfaces overnight (**d**) and quantification (**e**). *n* = 5 fields in each group; the data are presented as the mean ± s.d. values. Soft agar cultures of U2OS, MG63, HOS, and 143B cells for 2 weeks (**f**) and quantification (**g**, **h**). *n* = 5 fields in each group; the data are presented as the mean ± s.d. values. The graphs show the individual data points derived from three independent measurements and the means. **P < 0.01; ***P < 0.001; ****P < 0.000 1; ^##^*P* < 0.01; ^###^*P* < 0.001. Scale bars, 50 μm

Rigidity-sensing modules are essential for cell contraction, and contractions of normal cells are typically short-lived with rapid disassembly of adhesions on soft surfaces, which can lead to cell death through anoikis.^6^ Therefore, we sought to examine whether the mechanosensing function of transformed OS cells is compromised. U2OS, MG63, HOS, and 143B cells were seeded on glass surfaces, and decreased focal adhesion (FA) areas and cytoskeleton strength were observed in HOS and 143B cells (Fig. 2a–c). Additionally, we synthesized hydrogels with varying stiffnesses to evaluate cell polarization in the OS cell lines. Notably, on soft surfaces, the aspect ratios of U2OS and MG63 cells were significantly diminished, while no noticeable differences were found in the aspect ratio of HOS or 143B cells (Fig. 2d, e). Clonal growth in soft agar is widely accepted to represent the classical phenotype of transformed cells.^6^ Thus, we cultured the OS cell lines in soft agar for 2 weeks, and colonies of HOS and 143B cells formed (Fig. 2f–h). Collectively, our results suggest that the rigidity sensing function is impaired in transformed OS cell lines (HOS and 143B cells).

### Toggling between normal and transformed growth based on sensor proteins

**Fig. 3:**
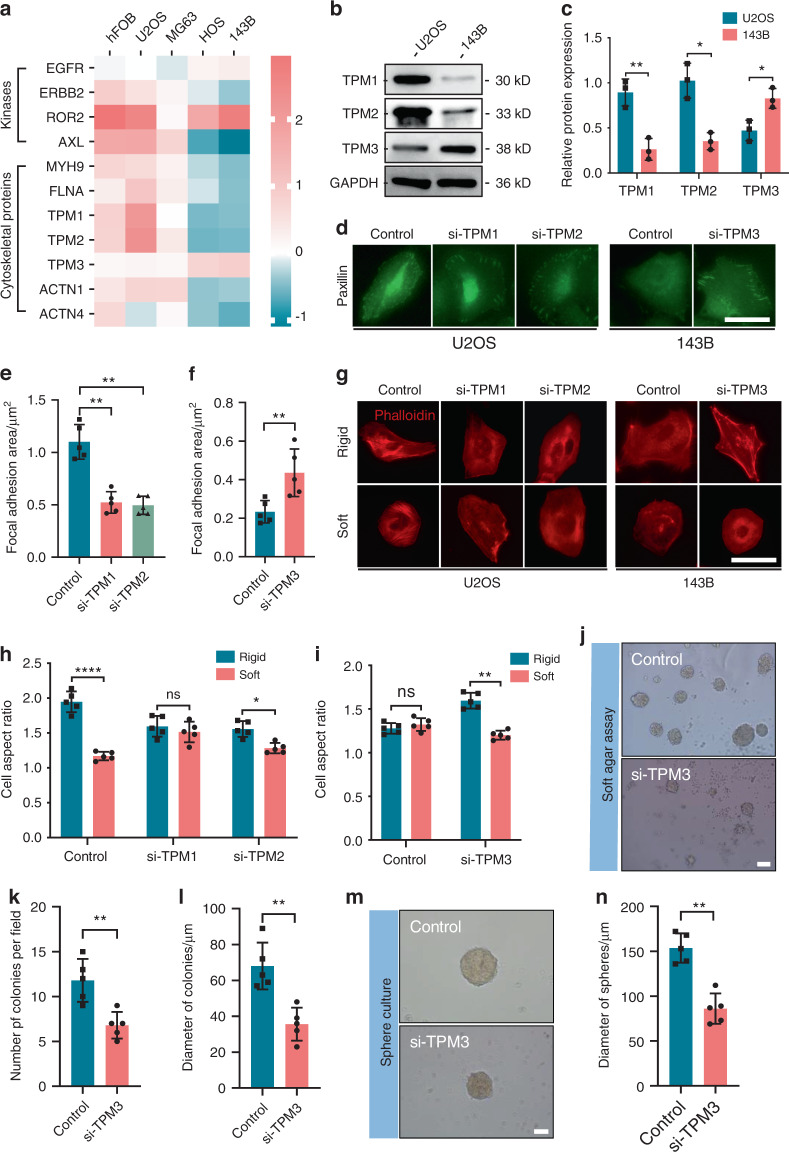
Toggling between normal and transformed growth based on sensor proteins. **a** Reductions in sensor protein expression levels in transformed cells (HOS and 143B) compared to nontransformed cells (hFOB, U2OS, and MG63), as determined by quantitative proteomics. *n* = 3 in each group; the data are presented as the means. Representative Western blots of sensor proteins (TPM1, TPM2, and TPM3) in U2OS and 143B cells (**b**) and quantification (**c**). *n* = 3 in each group; the data are presented as the mean ± s.d. values. Staining for paxillin (green) in *TPM1*- and *TPM2*-silenced U2OS cells and *TPM3*-silenced 143B cells cultured on glass surfaces overnight (**d**) and quantification (**e**, **f**). *n* = 5 fields in each group; the data are presented as the mean ± s.d. values. Staining for actin (red) in *TPM1*- or *TPM2*-silenced U2OS cells and *TPM3*-silenced 143B cells cultured on rigid (40 kPa) and soft (4 kPa) hydrogel surfaces overnight (**g**) and quantification (**h**, **i**). *n* = 5 fields in each group; the data are presented as the mean ± s.d. values. Soft agar cultures of *TPM3*-silenced 143B cells for 2 weeks (**j**) and quantification (**k**, **l**). *n* = 5 fields in each group; the data are presented as the mean ± s.d. values. Sphere cultures of *TPM3*-silenced 143B cells propagated for 7 days (**m**) and quantification (**n**). *n* = 5 fields in each group; the data are presented as the mean ± s.d. values. The graphs show the individual data points derived from three independent measurements and the means. *P < 0.05; **P < 0.01; ****P < 0.000 1. Scale bars, 50 μm

The rigidity-sensing proteins reported thus far comprise four kinases and seven cytoskeletal proteins. We sought to explain the impaired rigidity sensing function of OS cells by quantifying the expression levels of sensor proteins. By quantitative proteomics, we found reductions in the levels of sensor-related proteins (ERBB2, AXL, MYH9, FLNA, TPM1, TPM2, ACTN1, and ACTN4) but increased levels of EGFR and TPM3 in HOS and 143B cells (Fig. 3a). Western blot analysis further confirmed the decreased levels of TPM1 and TPM2 but increased level of TPM3 in 143B cells (Fig. 3b, c). Since tropomyosin is an important component of the mechanosensing machinery, controlling sarcomere-like contraction and suppressing growth on a soft matrix in normal cells,^8^ we selected U2OS cells, with high TPM1 and TPM2 expression, and 143B cells, with high TPM3 expression, for further experiments. Following the depletion of endogenous *TPM1* or *TPM2* in U2OS cells (Fig. S1a–d), the FA area was significantly reduced on glass surfaces (Fig. 3d–f). The polarization of U2OS cells was suppressed on rigid surfaces but promoted on soft surfaces (Fig. S2a), indicating impaired rigidity sensing (Fig. 3h). To test whether TPM3 suppresses rigidity sensing, we silenced endogenous *TPM3* in 143B cells (Fig. S1e, f). The FA area was significantly increased after this modification (Fig. 3d–f). The polarization of 143B cells was promoted on rigid surfaces but suppressed on soft surfaces (Fig. S2b), indicating restored rigidity sensing (Fig. 3i). Furthermore, the transformed growth of 143B cells was significantly suppressed in soft agar assays (Fig. 3j–l) and sphere cultures (Fig. 3m, n) after depletion of endogenous *TPM3*. Our data reveal toggling between normal and transformed growth in OS cell lines based on the expression pattern of rigidity-sensing proteins.

### Rigidity-sensing proteins were not regulated by *YAP1*

**Fig. 4:**
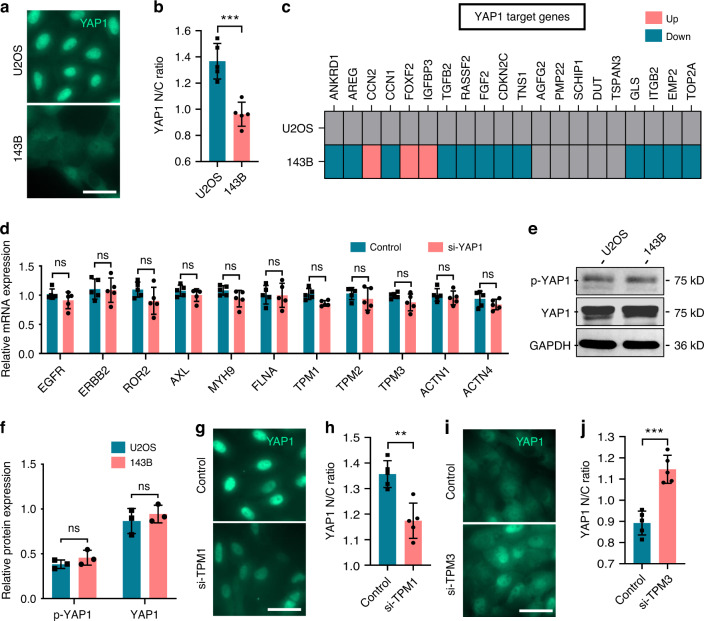
Sensor proteins mediate the subcellular localization of YAP1. Staining for YAP1 (green) in U2OS and 143B cells cultured on glass surfaces (**a**) and quantification of YAP1 localization (**b**). *n* = 5 fields in each group; the data are presented as the mean ± s.d. values. **c** Expression of YAP1 target genes in U2OS and 143B cells cultured on glass surfaces. *n* = 3 in each group. **d** Expression of rigidity-sensing genes in *YAP1*-silenced U2OS cells. *n* = 5 in each group; the data are presented as the mean ± s.d. values. Representative Western blots of YAP1 and p-YAP1 in U2OS and 143B cells cultured on glass surfaces (**e**) and quantification (**f**). *n* = 3 in each group; the data are presented as the mean ± s.d. values. Staining for YAP1 (green) in *TPM1*-silenced U2OS cells cultured on glass surfaces (**g**) and quantification of YAP1 localization (**h**). *n* = 5 fields in each group; the data are presented as the mean ± s.d. values. Staining for YAP1 (green) in *TPM3*-silenced 143B cells cultured on glass surfaces (**i**) and quantification of YAP1 localization (**j**). *n* = 5 fields in each group; the data are presented as the mean ± s.d. values. The graphs show the individual data points derived from three independent measurements and the means. **P < 0.01; ***P < 0.001. Scale bars, 50 μm

We attempted to predict the upstream transcription factors of rigidity-sensing proteins using ChEA3, and TEAD1, TEAD3, and TEAD4 were identified (Fig. S3). Mechanistically, YAP1 can bind to TEADs (TEAD1-4) and then promote the expression of target genes, including regulators of the cell cycle, cell migration, and cell fate.^23^ We observed a reduction in the YAP1 nucleocytoplasmic (N/C) ratio in 143B cells compared to U2OS cells, and 12 of the 20 YAP1 target genes were downregulated (Fig. 4a–c), indicating a lack of YAP1 activation in 143B cells. Therefore, we sought to determine whether these rigidity-sensing proteins are downstream of *YAP1*. Upregulation of sensor-related proteins (ERBB2, AXL, MYH9, FLNA, TPM1, TPM2, ACTN1, and ACTN4) was found in U2OS cells by quantitative proteomics. After silencing endogenous *YAP1* in U2OS cells (Fig. S1g, h), we did not observe a reduction in the expression of rigidity-sensing genes (Fig. 4d). Moreover, the levels of p-YAP1 and YAP1 were comparable in U2OS and 143B cells cultured on glass surfaces (Fig. 4e, f). However, the YAP1 N/C ratio was significantly reduced in *TPM1*-depleted U2OS cells (Fig. 4g, h) and significantly increased in 143B cells with *TPM3* knockdown (Fig. 4i, j). Taken together, our data demonstrate that rigidity-sensing proteins are not regulated by *YAP1* but that the nuclear distribution of YAP1 is driven by rigidity-sensing proteins.

### GOF mutation of *TP53* in transformed OS cell lines

**Fig. 5:**
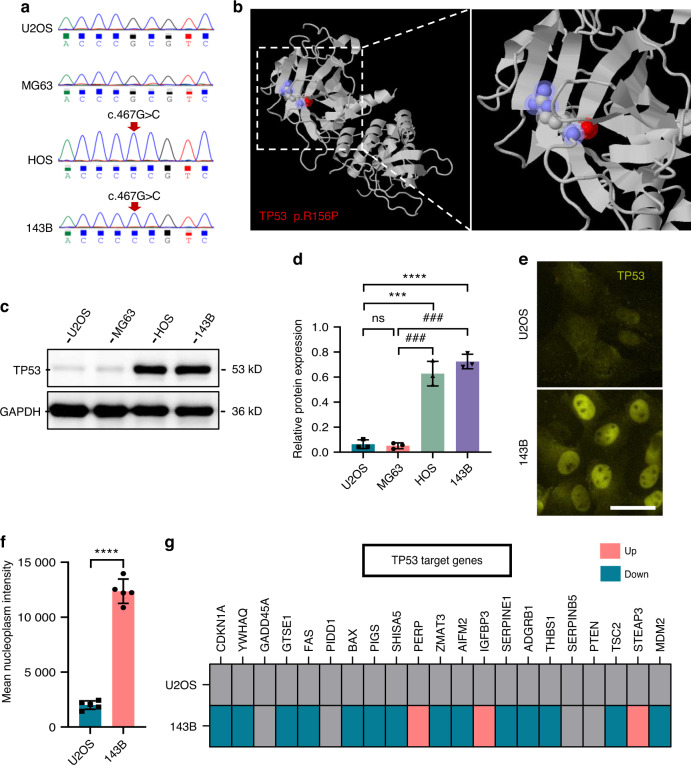
Mutant *TP53* in transformed osteosarcoma cell lines. **a** *TP53* mutations in HOS and 143B cells were identified using Sanger sequencing. **b** Representative structure of amino acid substitutions (red) in the mutant *TP53* protein in HOS and 143B cells. Representative Western blots of TP53 in U2OS, MG63, HOS, and 143B cells (**c**) and quantification (**d**). *n* = 3 in each group; the data are presented as the mean ± s.d. values. Staining for TP53 (yellow) in U2OS and 143B cells cultured on glass surfaces (**e**) and quantification (**f**). *n* = 5 fields in each group; the data are presented as the mean ± s.d. values. **g** Expression of TP53 target genes in U2OS and 143B cells cultured on glass surfaces. *n* = 3 in each group. The graphs show the individual data points derived from three independent measurements and the means. ***P < 0.001; ****P < 0.000 1; ^###^*P* < 0.001. Scale bars, 50 μm

Accumulating evidence has shown that mutations in *TP53* can promote cancer cell motility, invasion, and metastasis.^24^ Interestingly, the ChEA3 prediction also identified TP53 as one of the upstream transcription factors for sensor proteins (Fig. S3). The 20 genes with the highest mutation frequency in OS patients were obtained from the COSMIC database, and *TP53* was found to have the highest mutation frequency, 25% (Fig. S4). Therefore, we investigated the potential role of *TP53* in OS cell lines. Surprisingly, the *TP53* mutation (c.467G > C) in HOS and 143B cells was confirmed by Sanger sequencing (Fig. 5a). This missense mutation (R156P) was located in the *TP53* DNA-binding domain (Fig. 5b), and it was predicted to be probably damaging based on the HumDiv and HumVar models (Fig. S4a). By prediction of the TP53 structure using AlphaFold, we observed a hydrogen bond between ARG 156 and GLU 204 (Fig. S4b), suggesting that the missense mutation (R156P) in *TP53* may affect its secondary protein structure. In comparison to those in U2OS and MG63 cells, the TP53 protein levels were more than 100-fold higher in HOS and 143B cells (Fig. 5c, d), and the intensity of nucleoplasmic TP53 was also significantly increased in 143B cells (Fig. 5e, f). Notably, the accumulated TP53 protein in 143B cells did not activate but instead inhibited the expression of its downstream target genes (Fig. 5g).

Although the exact mechanisms of mutant TP53 reactivation remain to be elucidated, APR-246 has been shown to be effective in overcoming GOF activity by restoring wild-type TP53 function.^25^ We then verified the GOF of mutant *TP53* (R156P) in transformed OS cells by treatment with APR-246. Although the expression level of the TP53 protein was unchanged, the viability of 143B cells with mutant *TP53* was significantly reduced after treatment with APR-246, but no adverse effect was observed on U2OS cells with wild-type *TP53* (Fig. S5a–c). Furthermore, APR-246 treatment significantly inhibited the growth of 143B cells in soft agar assays (Fig. S5d, e), floating cultures (Fig. S5f, g), and xenograft mice (Fig. S5h, i). Collectively, our data demonstrate the presence of the *TP53* (R156P) mutation in HOS and 143B cells and reveal its GOF in these OS cell lines.

### Mutant *TP53* inhibits rigidity sensing in transformed OS cells

**Fig. 6:**
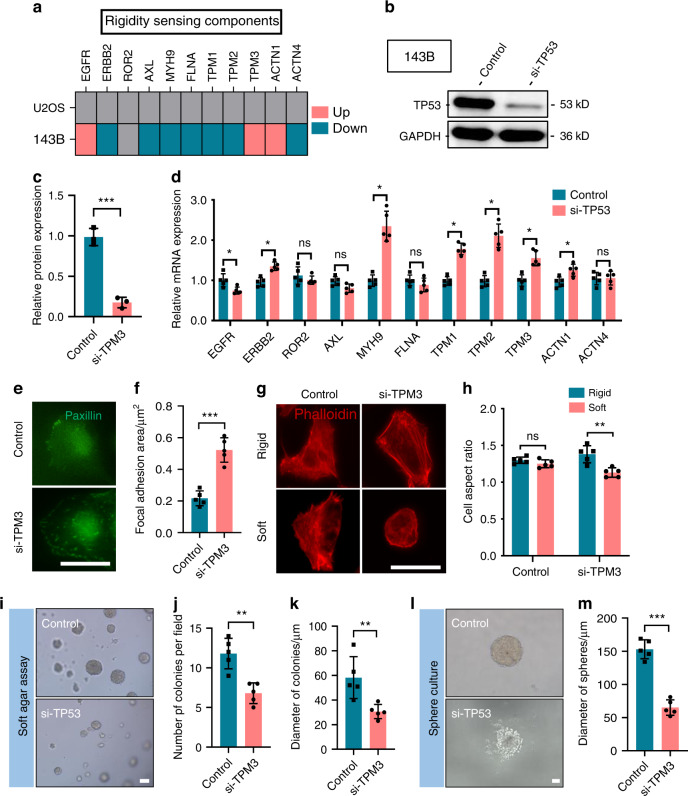
Mutant *TP53* inhibits rigidity sensing in transformed osteosarcoma cells. **a** Expression of rigidity-sensing genes in U2OS and 143B cells. *n* = 3 in each group. Representative Western blots of *TP53*-silenced 143B cells (**b**) and quantification (**c**). *n* = 3 in each group; the data are presented as the mean ± s.d. values. **d** Expression of rigidity-sensing genes in *TP53*-silenced 143B cells. *n* = 5 in each group; the data are presented as the mean ± s.d. values. Staining for paxillin (green) in *TP53*-silenced 143B cells (**e**) and quantification (**f**). *n* = 5 fields in each group; the data are presented as the mean ± s.d. values. Staining for actin (red) in *TP53*-silenced 143B cells cultured on rigid (40 kPa) or soft (4 kPa) hydrogel surfaces overnight (**g**) and quantification (**h**). *n* = 5 fields in each group; the data are presented as the mean ± s.d. values. Soft agar cultures of *TP53*-silenced 143B cells for 2 weeks (**i**) and quantification (**j**, **k**). *n* = 5 fields in each group; the data are presented as the mean ± s.d. values. Sphere cultures of *TP53*-silenced 143B cells propagated for 7 days (**l**) and quantification (**m**). *n* = 5 fields in each group; the data are presented as the mean ± s.d. values. The graphs show the individual data points derived from three independent measurements and the means. *P < 0.05; **P < 0.01; ***P < 0.001. Scale bars, 50 μm

Considering the GOF of mutant *TP53* in transformed OS cell lines, we next investigated its potential role in regulating rigidity-sensing proteins. We observed a significant reduction in the expression of rigidity-sensing genes (*ERBB2*, *AXL*, *MYH9*, *FLNA*, *TPM1*, *TPM2*, *ACTN1*, and *ACTN4*), with the exception of *EGFR* and *TPM3*, in 143B cells, as demonstrated by RNA sequencing (Fig. 6a). Following depletion of endogenous *TP53* in 143B cells, we found significantly increased expression of rigidity-sensing genes, including *ERBB2*, *MYH9*, *TPM1*, *TPM2*, *TPM3*, and *ACTN1*, as well as suppression of the previously upregulated expression of *EGFR* (Fig. 6b–d). In *TP53*-depleted 143B cells, the FA area was significantly increased on glass surfaces (Fig. 6e, f), and the cell aspect ratio was significantly decreased on soft surfaces (Figs. 6g, h and S2c). Moreover, depletion of *TP53* in 143B cells inhibited their transformed growth in soft agar (Fig. 6i–k) and sphere cultures (Fig. 6l, m). Collectively, these results support the idea that mutant *TP53* inhibits rigidity sensing in transformed OS cells, whereas depletion of endogenous *TP53* in 143B cells restores rigidity sensing, thus inhibiting transformed growth. In summary, our data reveal a novel GOF of mutant *TP53* in inhibiting the rigidity sensing function of transformed OS cells.

## Discussion

The origin of the heterogeneity of OS has remained unclear, and changes in tumorigenicity and mechanotransduction have been the focus of intense interest. Our findings indicate that rigidity sensing plays a fundamental role in OS tumorigenicity as an element in cellular sensing of the physical microenvironment. Notably, our results showed that the rigidity sensing function of transformed OS cells was impaired and that transformed growth could be modulated based on the expression patterns of sensor proteins. We found that the mechanotransduction protein YAP1 did not directly regulate rigidity-sensing molecules, whereas the nuclear distribution of YAP1 was regulated by rigidity sensing proteins. Mechanistically, we identified a novel *TP53* mutation in transformed OS cells that resulted in GOF to inhibit the rigidity sensing function, thereby maintaining transformed growth.

The tumorigenicity of OS cell lines used in basic research varies greatly, and the underlying reasons for the differences in tumorigenicity among OS cell lines remain unclear.^4^ We tested the tumorigenicity of OS cell lines in vitro and in vivo, and anoikis resistance was found in OS cell lines (143B and HOS cells) with aggressive phenotypes. Pulmonary metastasis of OS is commonly observed and is attributed to anoikis resistance in OS cells, as reported in previous studies.^26^ The absence of normal signaling generated by attachment to the ECM could constitute a driving force for anoikis resistance.^11^ Functionally, OS cells showed different rigidity sensing patterns, including differences in the focal adhesion area, cytoskeletal force, cell shape, and anchorage-independent growth, upon culture on rigid or soft surfaces. The impact of substrate stiffness on OS cell spreading and aggregation has been previously investigated, and MG63 and U2OS cells were found to form multicellular aggregates when cultured on soft substrates.^27^ Similarly, another study reported that MG63 cells were sensitive to ECM adhesion, similar to observations in human fetal osteoblastic (hFOB) cells in both 2D and 3D culture.^28^ In this study, the rigidity sensing function was shown to prevent the tumorigenic growth of U2OS and MG63 cells on soft matrix surfaces or under nonadherent conditions. This suggests that great caution needs to be taken when choosing cell lines for evaluation in Matrigel-based 3D culture models or in vivo studies. Moreover, our results reveal that the rigidity sensing function of transformed OS cells is impaired, and it is of great importance to study the molecular mechanisms of these aggressive OS cells.

Many kinases and cytoskeletal proteins are involved in the formation of rigidity-sensing components.^6^ We sought to explain the impaired rigidity sensing function of OS cells by quantifying the expression of sensor proteins. The expression patterns of sensor proteins in U2OS and MG63 cells were similar to those in hFOB cells, whereas the expression levels of most sensor proteins (ERBB2, AXL, MYH9, FLNA, TPM1, TPM2, ACTN1, and ACTN4) were decreased in 143B and HOS cells. In epithelial cells, MYH9 and TPM2 are required for the binding of vinculin to E-cadherin in response to rigidity and tension.^29^ However, the absence of rigidity-sensing components in many cancer cells could facilitate their spread in soft environments. The brain is a very soft tissue, and TPM2 loss has been shown to promote the colonization of primary patient-derived glioblastoma cells on soft substrates.^30^ Rigidity-sensing modules have been analyzed by immunoblotting in many transformed cell lines, including the human breast cancer cell line MDA-MB-23, the fibrosarcoma cell line HT1080, and the mouse lung carcinoma cell line LCC. All these transformed cell lines lacked at least one rigidity-sensing module.^6^ In addition, both TPM1 and FLNA have been identified as independent protective factors for OS patient survival in different prognostic risk models.^1,31^ Our data reveal toggling between normal and transformed growth in OS cell lines following depletion of endogenous *TPM1*, *TPM2*, and *TPM3*, and this toggling is dependent on the expression of rigidity sensor proteins. Interestingly, we found that TPM3 was highly expressed in HOS and 143B cells and suppressed rigidity sensing. Reduced expression of TPM2 and increased expression of TPM3 have been reported in cells transformed by various oncogenes.^32^ TPM2 is copolymerized on the same actin filaments as TPM3, and competition between TPM2 and TPM3 has been reported to occur in human foreskin fibroblast cells.^6^ Similarly, overexpression of TPM3 in mouse muscle was found to reduce the expression of endogenous TPM2 and, to a lesser degree, TPM1.^33^ Therefore, these important sensing proteins are promising therapeutic targets for OS.

*TP53* is the most frequently altered gene in OS, and our data reveal a novel mutation (R156P) and verify the GOF of the corresponding mutant in HOS and 143B cells. In a mouse model of Li-Fraumeni syndrome, an increased incidence of metastatic osteosarcomas was found in heterozygous mice with an endogenous mutant allele of *TP53* (corresponding to the R127H mutation).^34^ In comparison to parental MG63 cells, MG63 cells with ectopic expression of a *TP53* mutant (R248W/P72R) exhibited enhanced sphere formation and clonogenic growth, indicating that this GOF could be the root cause of the dedifferentiation of MG63 cells into cancer stem cells.^35^ We found abnormal accumulation of the TP53 protein in HOS and 143B cells compared with U2OS and MG63 cells. This finding was supported by the observation that the expression of TP53 was negatively correlated with the apoptotic index in OS tissues, and TP53 expression could be used as a potential biomarker for predicting the progression and prognosis of OS.^36^ Surprisingly, we observed increased expression of rigidity-sensing genes after depletion of endogenous *TP53* in 143B cells, and these results reveal a GOF of mutant TP53 in regulating rigidity-sensing proteins. Of note, *TMP3* mRNA expression was further increased after *TP53* knockdown. The possible reason for this phenomenon could be the specific regulatory effect of mutant TP53 on kinases (EGFR and ERBB2) in transformed OS cells. The ERBB signaling pathway is a large and complex system that regulates the downstream RAS/MAPK, AKT, JAK/STAT, and Src/FAK signaling axes involved in tumor cell migration, and *TPM3* is a component of the “pathways in cancer” network (KEGG: map05200).^37^ In particular, the Src/FAK signaling axis plays an important role in local adhesion and stiffness sensing processes.^38^ The potential feedback loop between mutant *TP53* and the ERBB signaling pathway warrants further exploration. The importance of cell rigidity sensing in controlling normal growth has been highlighted.^29^ However, the depletion of rigidity-sensing modules is an enabling factor for cancer progression even under attachment loss conditions.^7^ For the first time, our results revealed a novel GOF mutation of *TP53*, which may be the root cause of the rigidity sensing dysfunction in OS cells. These findings suggest the utility of universal screening for *TP53* variants in OS patients and the development of precision cancer therapies targeting oncogenic *TP53*.

In conclusion, our findings indicate a fundamental role of rigidity sensing components in OS tumorigenicity as mechanotransduction elements in cellular sensing of the physical microenvironment. The rigidity sensing function was impaired in transformed OS cell lines, and transformed growth could be modulated based on the expression patterns of rigidity sensing proteins. Mechanistically, we identified a novel *TP53* mutation in transformed OS cell lines that resulted in GOF to inhibit the expression of rigidity-sensing proteins, thereby maintaining transformed growth. Given its powerful oncogenic activity, the GOF mutant of TP53 may serve as an executor of these malignant programs.

## Materials and methods

### Cell culture and assay

hFOB cells (Procell) were cultured at 34 °C in a 5% CO_2_ incubator in Dulbecco’s modified Eagle’s medium/F12 supplemented with 10% fetal bovine serum (FBS). 143B cells (BNCC) were cultured at 37 °C in a 5% CO_2_ incubator in Roswell Park Memorial Institute-1640 medium supplemented with 10% FBS. U2OS cells (Procell) were cultured at 37 °C in a 5% CO_2_ incubator in McCoy’s 5A medium supplemented with 10% FBS. MG63 cells (Procell) were cultured at 37 °C in a 5% CO_2_ incubator in MEM supplemented with 10% FBS.

For the sphere formation assay, single-cell suspensions (1 000 per well) were seeded in 96-well ultralow adherence plates (Corning) in N2B27-defined serum-free medium for 7 days. A 3D culture model with Matrigel (Absin) was established. Briefly, Matrigel was mixed with the cell suspension at a 1:1 volume after thawing on ice. Drops of the mixture were added to the center of a 24-well plate (50 μL of mixture containing 2 000 cells per well) and incubated at 37 °C for 10 min, and the cells were cultured in complete medium for 2 weeks. Floating culture was performed in 6-well ultralow adherence plates (Corning) with complete growth medium. The soft agar assay was performed in 6-well plates using two layers of agarose gel at different concentrations. To form the solidified layer, 0.5% agarose was mixed with growth medium and left at room temperature until the gel had set. To form the upper layer, 0.4% agarose was mixed with growth medium. The cell suspensions (10 000 per well) were then added to the abovementioned mixture and quickly added to the 6-well plate. After the upper agarose layer had solidified, OS cells were incubated on agar with a concentration corresponding to a Young’s modulus of <2 kPa for 2 weeks. After treatment with APR-246 (MCE), cell viability assays were performed in 96-well plates using the Cell Counting Kit-8 (Beyotime) according to the manufacturer’s instructions.

### In vivo tumorigenicity assay

Animal experiments were conducted according to protocols approved by the Experimental Animal Welfare Ethics Committee. hFOB cells as well as OS cell lines (U2OS, MG63, 143B, and HOS) were used for xenograft model establishment in BALB/c nude mice. Briefly, single-cell suspensions (1 × 10^6^ cells) in 100 μL of serum-free medium were injected subcutaneously into BALB/c-nu mice. To validate the gain of function of mutant *TP53*, 143B and HOS cells were injected subcutaneously into BALB/c-nu mice. Mice were randomized to treatment cohorts once the tumor volume was more than 150 mm^3^ and were then administered either vehicle (0.9% saline) or APR-246 (50 mg·kg^−1^ in 0.9% saline) via intraperitoneal injection. Injections were performed daily for 7 days. Tumor size was measured weekly, and tumor volume was calculated using the following formula: (length × width × height)/2. The mice were killed before the tumors had a volume of 1 500 mm^3^ or a major axis length of 20 mm. For the low tumorigenicity and nontumorigenic cell lines, the experiments were stopped after 5 weeks.

### Preparation of soft and rigid PEG hydrogel surfaces

To provide soft and rigid surfaces for cell culture, PEG hydrogel and peptide conjugates were prepared according to a previous report.^39^ PEG-MAL (5 mmol·L^−1^) and arginine-glycine-aspartate peptides (GCGYGRGDSSPG) were dissolved in phosphate-buffered saline (PBS) for 1 h at 37 °C. Arginine-glycine-aspartate peptides were covalently conjugated to the PEG-MAL backbone through Michael addition between the cysteine residues on the peptides and the maleimide on the PEG-MAL backbone. Then, different concentrations of a PEG-SH crosslinker were added to react with the dual peptide-modified PEG-MAL. Hydrogel stiffness could be tuned independently from 2 to 41 kPa by varying the concentration of PEG-SH, thus allowing the preparation of soft (4 kPa) and rigid (40 kPa) hydrogel surfaces for cell seeding.

### siRNA transfection and immunoblotting

After cells were seeded overnight, *TPM1*, *TPM2*, *TPM3*, *YAP1*, and *TP53* siRNA (Tsingke) and Lipofectamine RNAiMAX (Invitrogen) were mixed for transfection on the following day. Control cells were transfected with scrambled siRNA (Tsingke). The knockdown efficiency was verified on the 3rd day, and the cells were then used for further experiments. For immunoblotting, cells were lysed in radioimmunoprecipitation assay buffer supplemented with 1% phenylmethanesulfonyl fluoride, and protein quantification was performed using the bicinchoninic acid method. The extracted proteins were separated by 10% gel electrophoresis and transferred to polyvinylidene fluoride membranes. The membranes were incubated with the following primary antibodies at 4 °C overnight: anti-GAPDH (Proteintech, dilution 1:50 000), anti-BAX (Proteintech, dilution 1:2 000), anti-Caspase-3 (Proteintech, dilution 1:1 000), anti-Caspase-9 (CST, dilution 1:1 000), anti-TPM1 (Proteintech, dilution 1:1 000), anti-TPM2 (Proteintech, dilution 1:1 000), anti-TPM3 (Proteintech, dilution 1:500), anti-YAP1 (Santa Cruz, dilution 1:500), anti-p-YAP1 (CST, dilution 1:1 000), and anti-TP53 (Proteintech, dilution 1:1 000). Primary antibody binding was detected using enhanced chemiluminescence with appropriate horseradish peroxidase-conjugated secondary antibodies.

### Gene expression profile

For transcriptome analysis, cells were washed with PBS, and total RNA was isolated using RNAiso (Takara) and a total RNA extraction kit (JIANSHI), as determined by the manufacturer’s guidelines. The RNA concentration was measured with a NanoDrop 2000 spectrophotometer (Thermo Fisher Scientific), and 1 μg of total RNA was used for library preparation and loaded into an Illumina HiSeq instrument for RNA sequencing. After transcriptomes were mapped, gene expression profiles were quantified as fragments per kilobase of transcript per million mapped reads using RNA-Seq. Real-time quantitative PCR was also performed. HiScript II RT SuperMix (Vazyme) with gDNA wiper was used to synthesize cDNA, and ChamQ SYBR qPCR Master Mix (Vazyme) was added to quantify the relative expression of target genes in an ABI QuantStudio 3 machine (Thermo Fisher Scientific). The gene-specific primers are listed in Table S1.

Quantitative proteomic analysis was performed using the tandem mass tag method.^40^ Briefly, hFOB, U2OS, MG63, HOS, and 143B cells were lysed using SDT buffer (4% SDS, 1 mmol·L^−1^ DTT, 100 mmol·L^−1^ Tris-HCl (pH 7.6)). Protein quantification was performed using a bicinchoninic acid assay kit, and protein enzymolysis was carried out using a filter-assisted sample preparation technique. Labeling was performed according to the instructions of the tandem mass tag kit, and labeled samples were fractionated and digested to generate peptides. Each peptide was identified using a nano LC‒MS/MS instrument (Thermo Fisher Scientific) connected to an Easy nLC system. MS/MS spectra were searched using the MASCOT engine (version 2.2) installed in Proteome Discoverer 1.4.

### Fluorescence microscopy and analysis

Cells were fixed with 4% paraformaldehyde for 15 min and were then incubated with 0.5% Triton® X-100. The samples were then blocked with 1% bovine serum albumin in PBS for 1 h, incubated with primary antibodies against Paxillin (Proteintech, 1:200), YAP1 (Santa Cruz, dilution 1:100), and TP53 (Proteintech, dilution 1:100) at 4 °C overnight, and then incubated with secondary antibodies for 2 h at 37 °C. Fluorescence images were acquired and analyzed with Harmony High-Content Imaging and Analysis Software (PerkinElmer). The YAP1 N/C was defined as the ratio of the nuclear to the cytoplasmic concentration of YAP1 and assessed by dividing the mean fluorescence intensity in the nucleus by that in the cytoplasm after subtraction of background fluorescence. The cytoplasmic region was determined by generating a mask extending 0.5 μm from the nuclear boundary into the cytoplasm.^41^ The cytoskeleton was stained with phalloidin for 1 h at 37 °C, and texture properties were used to quantify the strength of the cytoskeleton in a cell region. The methods of analyzing texture features include SER feature extraction, Haralick feature extraction, and Gabor filtering. SER features are frequently the best method for classifying cells, and the SER ridge is calculated as the intensity of a corresponding filtered image averaged over the corresponding object. Quantification of SER features was performed according to the manufacturer’s guidelines of Harmony High-Content Imaging and Analysis Software (PerkinElmer).

### Sanger sequencing and protein structure prediction

DNA was extracted from U2OS, MG63, 143B, and HOS cells using the DNA Mini Kit (Qiagen). After PCR amplification, the *TP53* mutation (c.467G > C) was confirmed with Sanger sequencing using an ABI 3730 genetic analyzer (Thermo Fisher Scientific). The protein structure of TP53 was predicted with PolyPhen-2 (http://genetics.bwh.harvard.edu/pph2/index.shtml),^42^ and AlphaFold (https://www.alphafold.ebi.ac.uk).^43^ Prediction of transcription factors for rigidity sensing genes was performed with ChEA3 (https://maayanlab.cloud/chea3/).^44^ The frequency of *TP53* mutations in OS patients was derived from the COSMIC database (https://cancer.sanger.ac.uk/cosmic).^45^

### Statistical analysis

GraphPad Prism software (version 8.2.1) was used for data analysis and graphing. Analyses of significant differences were carried out using two-tailed Student’s *t* test or, for comparisons among more than two groups, the Mann–Whitney *U* test or one-way analysis of variance. The relative expression of proteins was calculated based on grayscale values using ImageJ software (version 1.52). The graphs show the individual data points derived from three independent measurements and the means, and a *P* value of <0.05 was considered to indicate a statistically significant difference.

## Supplementary information

Table S1

Fig. S1

Fig. S2

Fig. S3

Fig. S4

Fig. S5

Fig. S6

Supplementary Legend

## Data Availability

The data that support the findings of this study are available from the corresponding author upon reasonable request.
