# Supplementary material for: Impairment of rigidity sensing caused by mutant TP53 gain of function in osteosarcoma
Source: Bone Res. 2023 May 29;11:28. doi: 10.1038/s41413-023-00265-w (PMC10225464; doi:10.1038/s41413-023-00265-w)
Supplement: Supplementary file 1 — Table S1 [file 41413_2023_265_MOESM1_ESM.docx]

| **Gene** | **Primer** |
| --- | --- |
| *GAPDH*, F | GGAGCGAGATCCCTCCAAAAT |
| *GAPDH*, R | GGCTGTTGTCATACTTCTCATGG |
| *EGFR*, F | AGGCACGAGTAACAAGCTCAC |
| *EGFR*, R | ATGAGGACATAACCAGCCACC |
| *ERBB2*, F | TGTGACTGCCTGTCCCTACAA |
| *ERBB2*, R | CCAGACCATAGCACACTCGG |
| *ROR2*, F | TCCGAACGACCCTTTAGGAC |
| *ROR2*, R | TTTAGCCACCGCACGTTAGG |
| *AXL*, F | GTGGGCAACCCAGGGAATATC |
| *AXL*, R | GTACTGTCCCGTGTCGGAAAG |
| *MYH9*, F | CCTCAAGGAGCGTTACTACTCA |
| *MYH9*, R | CTGTAGGCGGTGTCTGTGAT |
| *FLNA*, F | CTTATCGCGCTGTTGGAGGT |
| *FLNA*, R | GCCACCGACACGTTCTCAA |
| *TPM1*, F | CGCAGAAGGAAGACAGATA |
| *TPM1*, R | CAAAACAAAACGAAAGAAT |
| *TPM2*, F | GAGAGCGAGAGAGGAATGA |
| *TPM2*, R | GGTCCAAGGTCTGGTGAAT |
| *TPM3*, F | TGAAAACCGGGCCTTAAAAGAT |
| *TPM3*, R | GATCACCAACTTACGAGCCAC |
| *ACTN1*, F | CCCGAGCTGATTGACTACGG |
| *ACTN1*, R | GCAGTTCCAACGATGTCTTCG |
| *ACTN4*, F | GCAGCATGGGCGACTACAT |
| *ACTN4*, R | TTGAGCCCGTCTCGGAAGT |

**Supplemental Table 1.** The real time quantitative PCR primers for human.
