# Supplementary figures and images for: Impairment of rigidity sensing caused by mutant TP53 gain of function in osteosarcoma
Source: Bone Res. 2023 May 29;11:28. doi: 10.1038/s41413-023-00265-w (PMC10225464; doi:10.1038/s41413-023-00265-w)

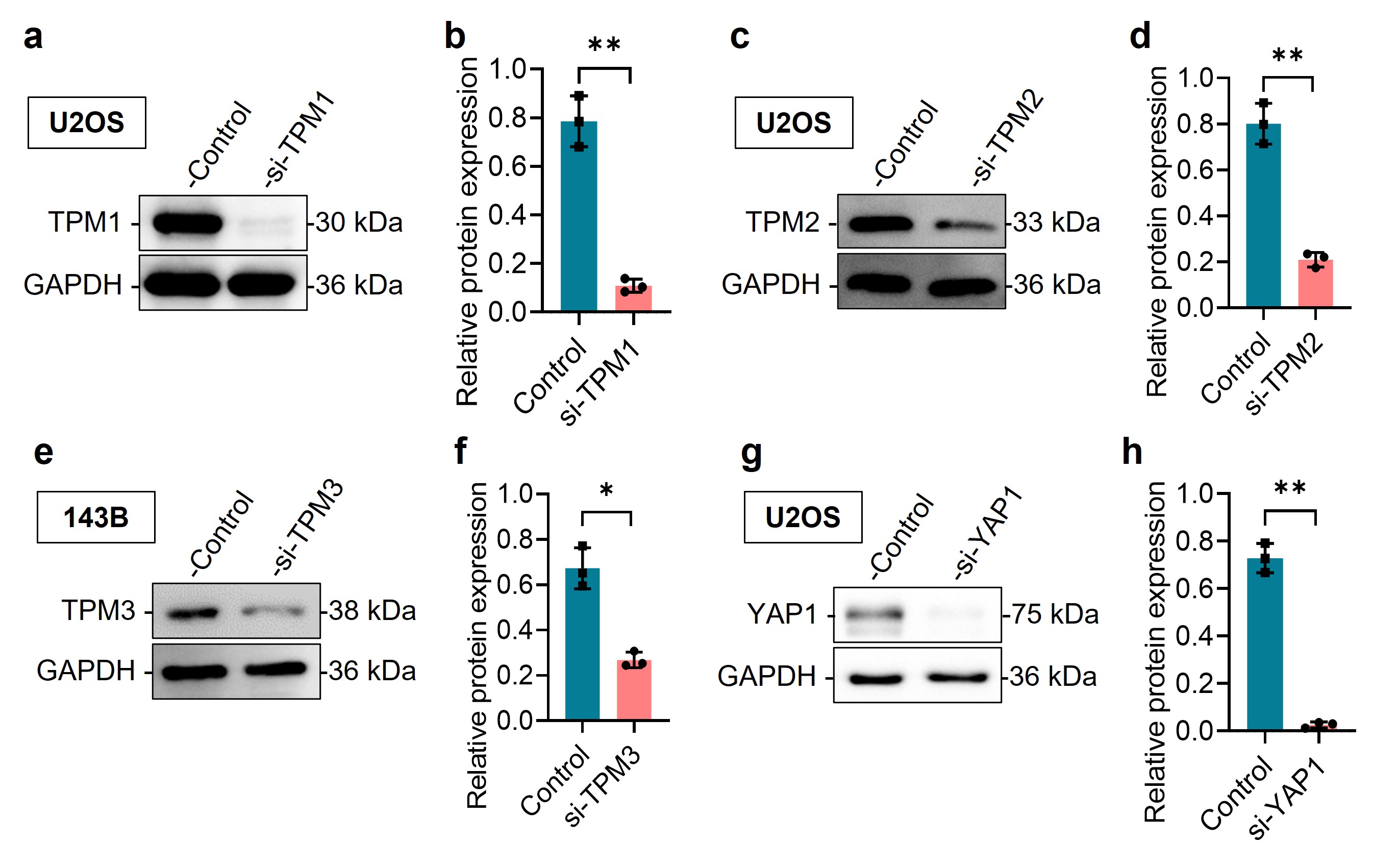

Supplement: Supplementary file 2 — Fig. S1 [file 41413_2023_265_MOESM2_ESM.jpg]

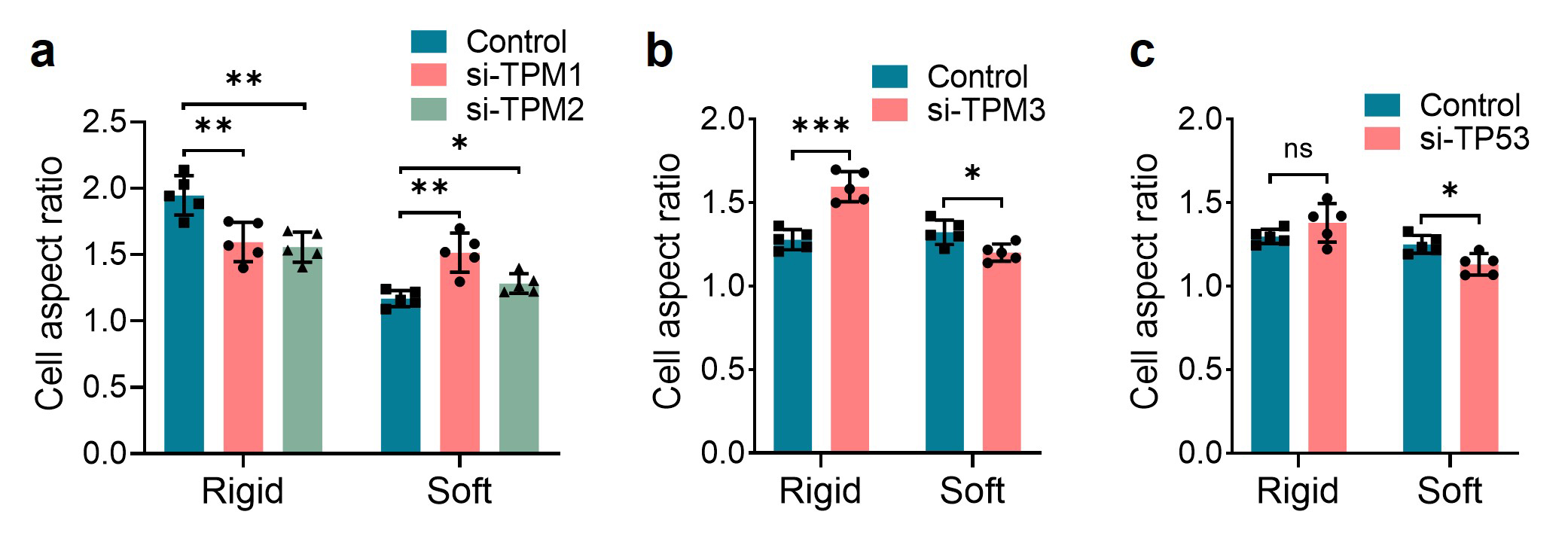

Supplement: Supplementary file 3 — Fig. S2 [file 41413_2023_265_MOESM3_ESM.jpg]

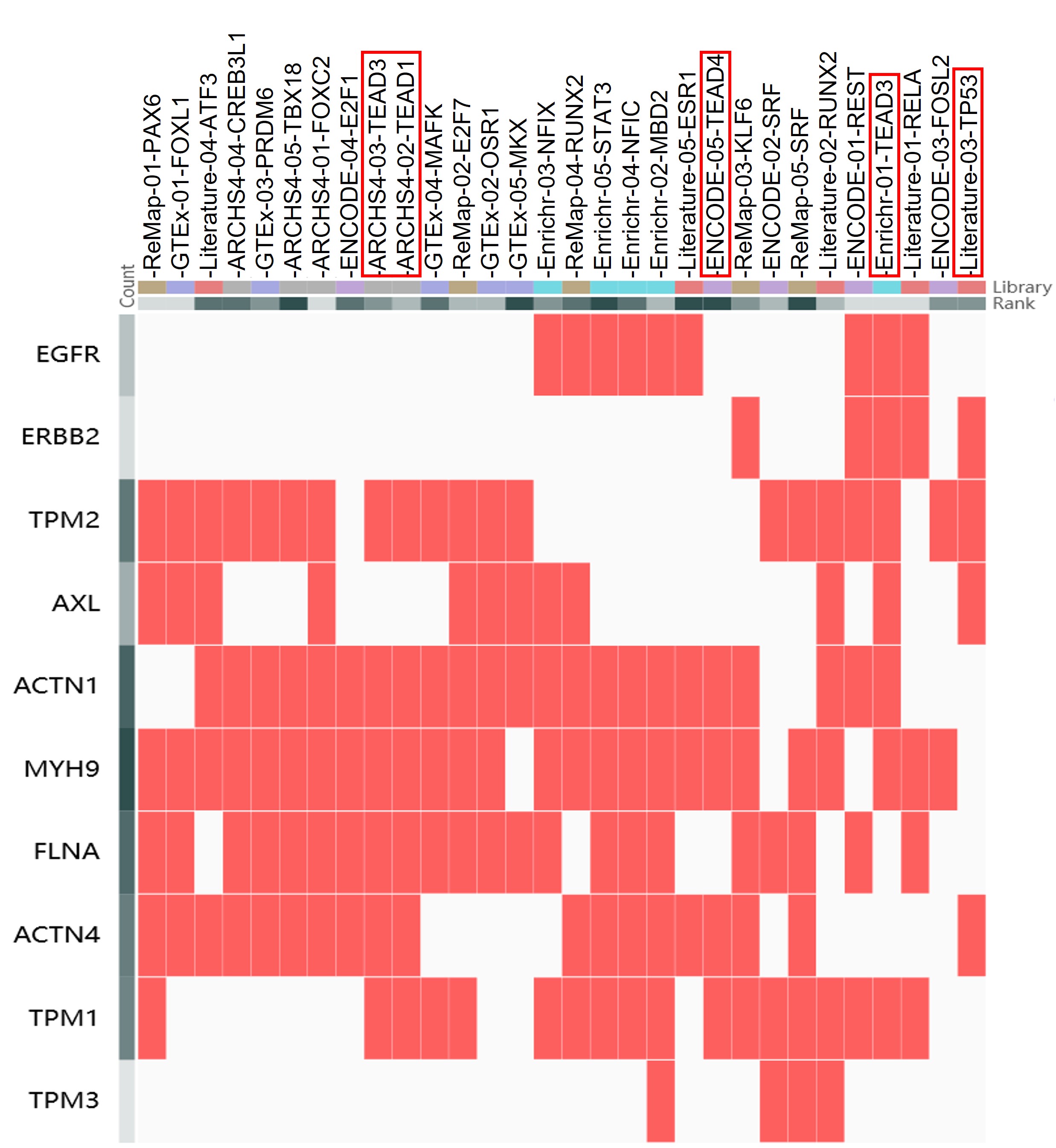

Supplement: Supplementary file 4 — Fig. S3 [file 41413_2023_265_MOESM4_ESM.jpg]

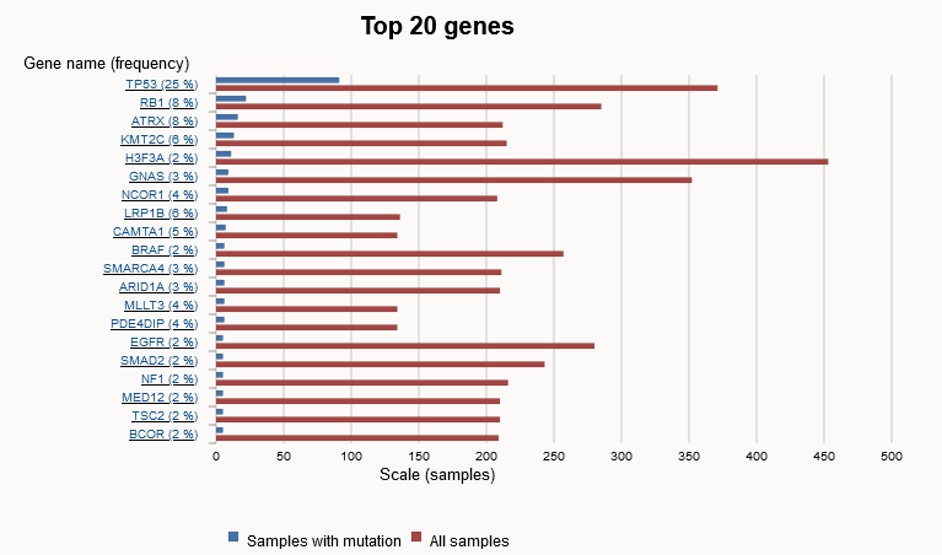

Supplement: Supplementary file 5 — Fig. S4 [file 41413_2023_265_MOESM5_ESM.jpg]

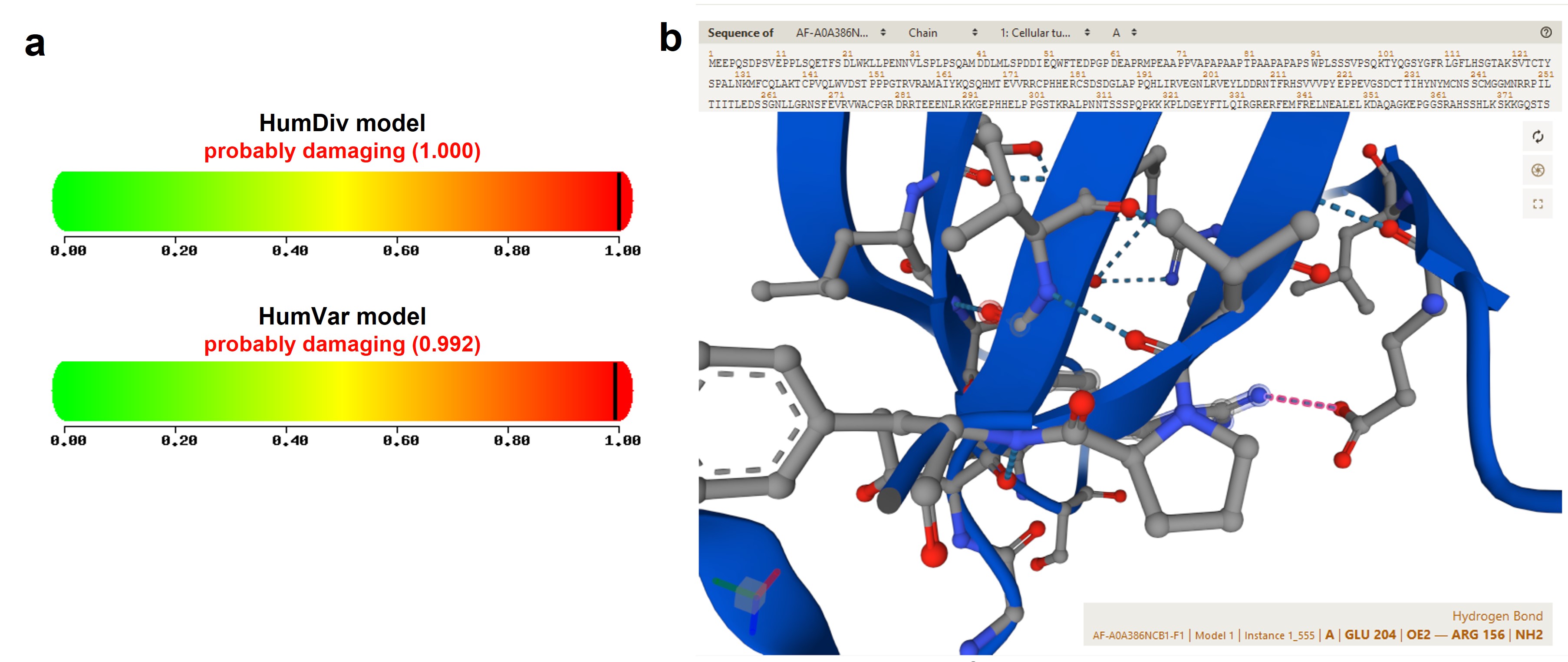

Supplement: Supplementary file 6 — Fig. S5 [file 41413_2023_265_MOESM6_ESM.jpg]

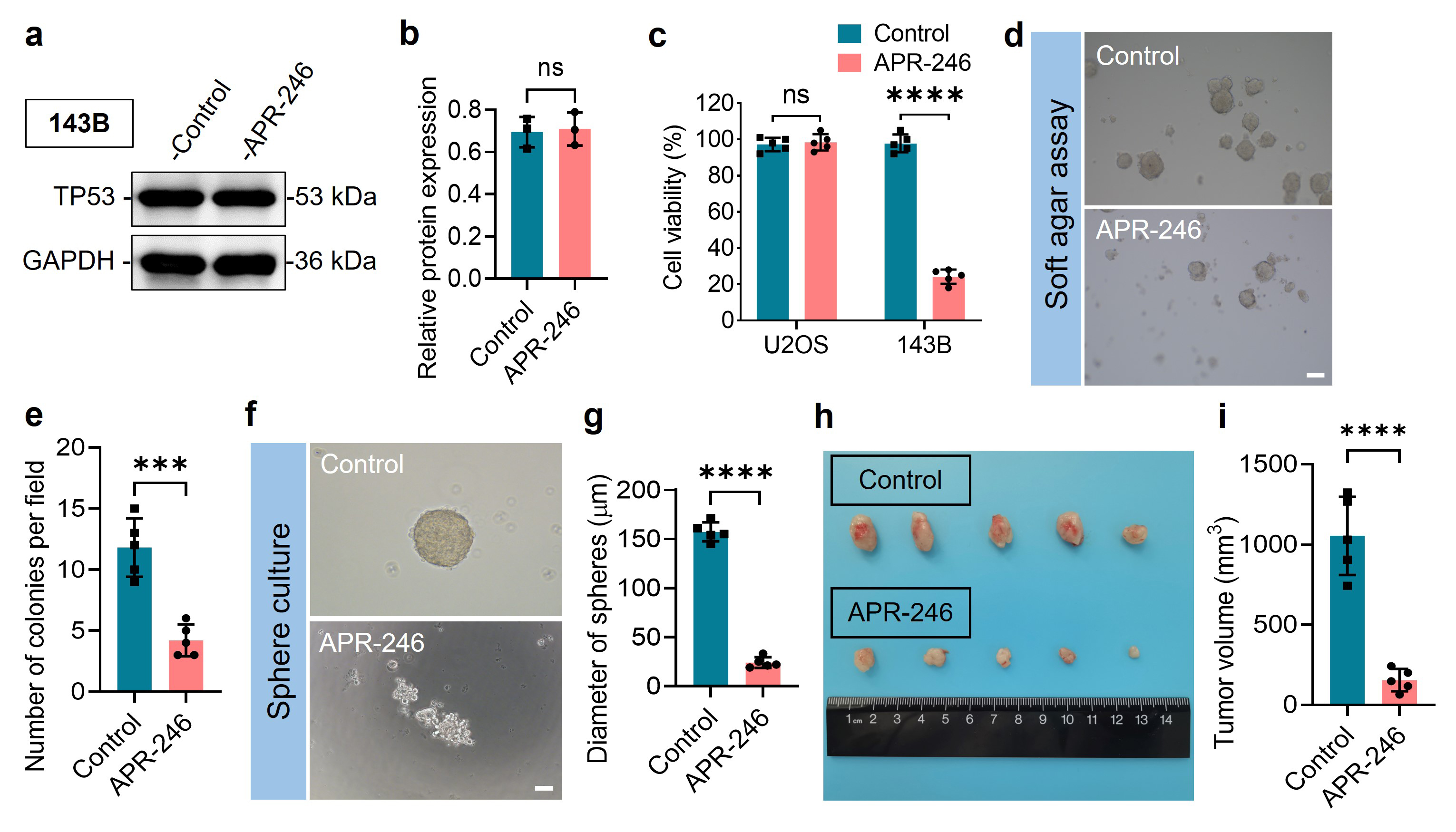

Supplement: Supplementary file 7 — Fig. S6 [file 41413_2023_265_MOESM7_ESM.jpg]
