# Supplementary material for: Impairment of rigidity sensing caused by mutant TP53 gain of function in osteosarcoma
Source: Bone Res. 2023 May 29;11:28. doi: 10.1038/s41413-023-00265-w (PMC10225464; doi:10.1038/s41413-023-00265-w)
Supplement: Supplementary file 8 — Supplementary Legend [file 41413_2023_265_MOESM8_ESM.docx]

**Fig. S1 Validation of knockdown efficiency in osteosarcoma cells**

**a**, **b** Representative Western blots showing *TPM1* silencing in U2OS cells (**a**) and quantification (**b**). n=3 in each group; the data are presented as the mean ± s.d. values. **c**, **d** Representative Western blots showing *TPM2* silencing in U2OS cells (**c**) and quantification (**d**). n=3 in each group; the data are presented as the mean ± s.d. values. **e**, **f** Representative Western blots showing *TPM3* silencing in 143B cells (**e**) and quantification (**f**). n=3 in each group; the data are presented as the mean ± s.d. values. **g**, **h** Representative Western blots of *YAP1* silencing in U2OS cells (**g**) and quantification (**h**). n=3 in each group; the data are presented as the mean ± s.d. values. The graphs show the individual data points derived from 3 independent measurements and the means. **P*<0.05; ***P*<0.01.

**Fig. S2 Rigidity-sensing proteins mediate cell polarization**

**a** Comparison of the cell aspect ratios of TPM1- and TPM*2*-silenced U2OS cells cultured on rigid (40 kPa) and soft (4 kPa) hydrogel surfaces overnight. n=5 in each group; the data are presented as the mean ± s.d. values. **b** Comparison of the cell aspect ratio of *TPM3*-silenced 143B cells cultured on rigid (40 kPa) and soft (4 kPa) hydrogel surfaces overnight. n=5 in each group; the data are presented as the mean ± s.d. values. **c** Comparison of the cell aspect ratio of *TP53*-silenced 143B cells cultured on rigid (40 kPa) and soft (4 kPa) hydrogel surfaces overnight. n=5 in each group; the data are presented as the mean ± s.d. values. The graphs show the individual data points derived from 3 independent measurements and the means. **P*<0.05; ***P*<0.01; ****P*<0.001.

**Fig. S3 Prediction of transcription factors for rigidity-sensing genes**

**Fig. S4 Top 20 genes mutated in osteosarcoma**

**Fig. S5 Pathogenicity and protein structure prediction**

**a** Pathogenicity prediction of the *TP53* mutant (R156P) using PolyPhen-2. **b** Protein structure prediction of wild-type TP53 using AlphaFold.

**Fig. S6 Gain of function of mutant *TP53* in osteosarcoma cells**

**a**, **b** Representative Western blots of proteins extracted from APR-246-treated 143B cells (**a**) and quantification (**b**). n=3 in each group; the data are presented as the mean ± s.d. values. **c** Cell viability assay of proteins extracted from APR-246-treated U2OS and 143B cells. n=5 in each group; the data are presented as the mean ± s.d. values. **d**, **e** Soft agar assays of 143B cells treated with APR-246 for 2 weeks (**d**) and quantification (**e**). n=5 fields in each group; the data are presented as the mean ± s.d. values. **f**, **g** Sphere cultures of APR-246-treated 143B cells propagated for 7 days (**f**) and quantification (**g**). n=5 fields in each group; the data are presented as the mean ± s.d. values. **h**, **i** Xenograft model established with APR-246-treated 143B cells (**h**) and quantification of tumor volume (**i**). n=5 in each group; the data are presented as the mean ± s.d. values. The graphs show the individual data points derived from 3 independent measurements and the means. ****P*<0.001; *****P*<0.0001. Scale bars, 50 μm.
